# Professor Charles A. Lee.—Biographical Sketch

**Published:** 1850-08

**Authors:** 


*Professor Charles A. Lee.—Biographical sketch.*—Under the title of "*Sketches of Eminent Living Physicians*," a writer in the Boston Medical and Surgical Journal, has given spirited and graphic biographical notices of several distinguished members of the medical profession in this country. The Sketches appear with the signature of *Cato.*

Our frend and colleague, Professor Lee, is the subject of the last sketch, (No. for the 10th ult.,) which we copy for the gratification of those of our readers, who desire to know something of the life, and peculiar traits of one whose name and writings are familiar to every one; and one who is honored and loved by his personal friends in proportion to the intimacy of friendship which it is their privilege to enjoy.

"The object of the following sketch, Charles A. Lee, M. D., was born about the year 1802, in the town of Salisbury, Ct. His father was a well-to-do farmer, and had been an officer in the revolutionary war. His grandfather, Rev. Jonathan Lee, is said to have been the first settled congregational minister in the above town. Until the age of 13 years Charles obtained his education in the common district schools, when, with the assistance of a private tutor, he began the study of the ancient languages. He afterwards became an inmate of the family of his uncle, Elisha Lee, Esq.. of Sheffield, Mass., who was an early graduate of Yale College, a distinguished classical scholar and advocate, a polished gentleman and a devout christian. After enjoying the benefits of his instruction in the Latin and Greek languages, and in English literature, he was sent to Lenox Academy, a celebrated school of learning, then under the charge of the eccentric Mr. Gleason. He continued here more than a year, prosecuting his studies with unwearied diligence; devoting in fact little time to the relaxation so necessary in growing boyhood. His health became materially injured by this continued devotion to study.

"In 1817 he entered Williams College, in Williamstown, Mass., in an advaced class, and graduated with distinguished honors in 1820. During his college studies his attention was by no means confined to the ordinary routine of the classes, but the Greek and Latin historians, as well as English literature and science, were eagerly devoured by him. He had also acquired that taste for the natural sciences which has so eminently distinguished his riper years. These studies were carried on under the direction Prof. C. Dewey. Another year spent in the city of New York, and we find him in the office of his brother-in-law, Dr. Luther Ticknor, of Salisbury, studying medicine. He graduated in 1825, after attending three full courses of lectures at in the medical institution at Pittsfield. The last two of these sessions he officiated as demonstrator of anatomy.

"After practicing a short time with Dr. Ticknor, he removed to New York city in 1826, and commenced the practice of his profession in earnest. He soon exhibited his spirit of enterprise by—in connection with Dr. James Stewart—founding the northern dispensary in that city. For four years he acted as attending physician, prescribing for more than 2000 patients per annum. He was, on retiring from these labors, appointed consulting physician, which office he continued to hold until quite recently, his private practice becoming too onerous to attend to the prescribing for so many public patients. His diligence in noting cases and making post-obit examinations was untiring. He found time, by way of relaxation, to prepare a popular and scientific work on Geology, for 'Harper's Family Library.' Another school-book from his pen, on Physiology, soon after appeared, both of which have had, and now have, a very extensive circulation. The latter, with those of Coates, Smith, and others, has tended to create the taste, now so general among the people, for physiological science. The Medical Journals were continually suppled with his cases, original articles, reviews and monographs—when indeed have they *not* been, since he began to wield his ever-moving pen! Among his monographs, were an elaborate essay on the Thymus Gland, on the Medical statistics of New York city, an Essay on the diseases of Clergymen, &c &c. &c. He also published a series of essays on the effects of the arts, trades and professions, on health and longevity, in the Eclectic Repository, of New York, which did much to awaken public attention to the subject of hygiene and medical police.

"In 1832 he was appointed, by the board of health, physician to the Greenwich Cholera Hospital, where he chiefly resided until the close of the epidemic. He was also physician to the New York Orphan's Asylum.

"In 1840 he originated the 'New York Journal of Medicine and the

Collateral Sciences,' associating with him the late lamented Dr. Forry ; to whom, in fact, in consequence of his other professional duties, he resigned the editorial chair, which was however resumed at the death of Dr. F. He never declined furnishing an article when requested, and indeed contributed freely to the Journal. He continued the editorship of the widely circulated Journal up to 1848. During all this time he wrote for its pages, attended to an extensive and increasing practice, and brought out in rapid succession, two large volumes of Copland's Medical Dictionary, with copious notes and additions ; and wrote some sixty lectures on General Pathology, and as many on Materia Medica. He edited Guy's Medical Jurisprudence, which he enlarged to nearly twice its original size. Paris' Pharmacologia and Thompson's Conspectus were edited, and nearly two-thirds of the matter in the last work was added, by Dr. Lee.

"In 1844 he was appointed Professor of General Pathology and Materia Medica in Geneva Medical College. Previous to this he had assisted in the organization of the medical department of the University of New York, and was appointed to the chair of Materia Medica and Therapeutics. He however declined before the institution went into operation.

"In 1847 he lectured in the Starling Medical College, at Columbus, Ohio ; and the same year was appointed to the chair of Materia Medica in Brunswick, (Maine,) Medical College, and in the University of Buffalo. His health having become impaired by these excessive labors, the intervals between the lecture terms, during the last three years, have been spent mostly in travelling. One summer was spent in the Lake country, exploring the whole copper regions of Lakes Superior, Huron, Michigan, &c. : his notes of which, are as yet unpublished. The summer of 1849 was spent in travelling over England, Ireland, Scotland, the Western Islands, and France ; a month was spent in Paris.

"Dr. Lee has a well selected library of three or four thousand volumes. His acquantance with American literature is perhaps as accurate as that of any medical writer in our country. He has an herbarium of some 1500 species of plants, collected with his own hands. His catalogue of the medicinal plants of the State of New York, is, and will continue to be, a monument to his industry and learning. The students of Geneva College will long remember his accuracy and kindness in explaining the plants which they gathered from day to day during the present spring session of that institution. He is an honorary member, we believe, of most of the scientific societies of this country and many of those of Europe.

"The mineralogy and geology of Salisbury received attention from Dr.

Lee in an article in Silliman's Journal. Pereira's celebrated work on food and diet, was edited by him, and received from his pen copious notes ; and what was very creditable to him, the copy right was secured and its entire avails awarded to the author in England.

"The temperance reform acknowledges in Dr. Lee a staunch friend and able defender. His pen has produced many able articles, which have been published in Boston, Albany, and New York city, on this subject. He has also delivered many public temperance addresses and lectures.

"To Dr. Lee is due, we believe, (at least we have seen no prior publication,) the credit of first openly advocating, in the pages of his journal, the subject of a National Medical Convention. The medical schools were at that time, as Cato pretty well knows, generally opposed to the measure, but have since come into it heartily. He also, long before any general movement was made in the matter, called the attention of the profession, in several articles on the subject, to the immense adulteration of drugs in the New York and other American markets. His devotion in fact, to everything calculated to elevate his beloved profession, is unremitting.

"As a lecturer, he is clear, terse and impressive. Having plenty of matter already written, he is at no loss for material, but is exceedingly happy when, throwing away his carefully written manuscript, he lets loose the current of his unharnessed thoughts. His humor is inexhaustible and irresistable, and he can, at will, chain the attention of the class by the expression of wit, pathos, literary and scientific anecdote, or instructive detail and narrative. He is, at once, respected and loved ; the poorest and most dependent student will confide in Dr. Lee, and the most accomplished will desire to learn from him. A consistent member of the Episcopal church, his religious character is as pure as his literary and scientific attainments are brilliant. About five feet eight and a half or nine inches in height, broad shoulders, with a strong frame ; hair of a *salt and pepper* grey, separated on one side of his forhead ; a large Roman nose, dark-blue eyes, and *a well-developed chin ;* a voice which is low and gentle ; manners quiet and rather retiring, more inclined to listen than to take the lead in conversation ; moderate and judicious in the expression of his opinions, and never violent or personal ; with a gait which may be denominated a *good long swing*—and we have Dr. Lee.

"Of such men, Cato thinks the medical profession of the United States, or of the world, ought to be proud—a true and beautiful specimen of what a physician ought to be. CATO."

